# Activation of ventral tegmental area vesicular GABA transporter (Vgat) neurons alleviates social defeat stress-induced anxiety in APP/PS1 mice

**DOI:** 10.3389/fnagi.2023.1142055

**Published:** 2023-03-23

**Authors:** Di Yao, Rong Li, Musa Kora, Hongqing Huang, Xinghua Liu, Song Gong

**Affiliations:** ^1^Trauma Centre/Department of Emergency and Trauma Surgery, Tongji Hospital, Tongji Medical College, Huazhong University of Science and Technology, Wuhan, Hubei, China; ^2^Department of Neurology, Tongji Hospital, Tongji Medical College, Huazhong University of Science and Technology, Wuhan, Hubei, China; ^3^Department of Pediatrics, Tongji Hospital, Tongji Medical College, Huazhong University of Science and Technology, Wuhan, Hubei, China; ^4^School of Basic Medicine, Tongji Medical College, Huazhong University of Science and Technology, Wuhan, Hubei, China

**Keywords:** Alzheimer’s disease, social defeat stress model, ventral tegmental area (VTA), GABA neuron, sleep

## Abstract

**Introduction:**

Alzheimer’s disease (AD) is a progressive neurodegenerative disease that results in cognitive impairment and is often accompanied by anxiety. In this study, we investigated whether the activation of VTA^Vgat^ neurons could reduce anxiety in APP/PS1 mice. We hypothesized that acute social defeat stress (SDS) would lead to anxiety in APP/PS1 mice, and that the activation of VTA^Vgat^ neurons would alleviate this anxiety.

**Methods:**

We exposed APP/PS1 mice to acute SDS and assessed anxiety using the open field test and elevated plus-arm test. Activated VTA^Vgat^ neurons was tested by cfos staining. Sleep quality was detected using electroencephalogram after SDS or non-SDS procedure. Sleep duration, sleep latency, and non-rapid eye movement (NREM) percentage were analyzed. VTA^Vgat^ neurons were chemogenetically activated by deschloroclozapine.

**Results:**

Our results showed that acute SDS led to anxiety in APP/PS1 mice, as evidenced by increased anxiety-related behaviors in the open field and elevated plus-arm tests. Activation of VTA^Vgat^ neurons by SDS led to an increase in sleep duration, primarily due to a decrease in sleep latency and an increase in NREMs. However, the quality of sleep was poor. Chemogenetical activation of VTA^Vgat^ neurons improved sleep quality and relieved SDS-induced anxiety. Furthermore, the anxiety state correlated negatively with sleep duration and NREM percentage and correlated positively with theta power density in APP/PS1 mice.

**Discussion:**

Our study provides evidence that the activation of VTA^Vgat^ neurons alleviates SDS-induced anxiety in APP/PS1 mice, suggesting that poor sleep quality may exacerbate anxiety in AD. These findings may have important implications for the treatment of anxiety in AD, as targeting VTA^Vgat^ neurons could be a potential therapeutic approach.

## Introduction

Alzheimer’s disease (AD) is a progressive neurodegenerative disease that manifests as cognitive dysfunction, which is often accompanied by anxiety (Pietrzak et al., 2015). Anxiety greatly interrupts the recovery of cognitive function, thus exacerbating the symptoms of amnesia. However, the mechanisms of anxiety associated with AD are not fully understood. Stress leads to anxiety and is therefore considered to be a key factor in the vicious cycle of anxiety-insomnia in AD patients. On the other hand, in rodents, social defeat stress (SDS) induces restorative sleep, which in turn alleviates anxiety (Yu et al., 2022). Thus, the stress-relieving effect of sleep would be one of the mechanisms that decelerates cognitive dysfunction. However, sleep is disturbed in patients with AD; hence, anxiety is not relieved.

Extracellular deposition of amyloid-β (Aβ) and intracellular neurofibrillary tangles are the two main pathological features of AD. Circadian oscillations in Aβ_42_ clearance suggest a link between sleep disturbance and Aβ burden (Clark et al., 2022). Sleep disorders are indeed associated with worse outcomes in older adults, such as increased Aβ burden, more symptoms of depression and cognitive decline (Winer et al., 2021). Positron emission tomography with ^18^F-florbetaben showed that one night of sleep deprivation induced a 5% increase in Aβ levels (Shokri-Kojori et al., 2018). In rodents, Aβ clearance in the brain interstitial fluid (ISF) occurs mainly during sleep, which is ascribed to the glymphatic pathway that operates most efficiently during sleep (Xie et al., 2013; Lee et al., 2015). Therefore, sleep disturbance is not only the seed of anxiety but also an accelerant of AD pathology.

The ventral tegmental area (VTA) regulates reward, aversion, and social contact (Yu et al., 2022). It also affects responses to stress and threats and strongly influences sleep and wakefulness: VTA vesicular glutamate transporter 2 (VTA^Vglut2^) and VTA tyrosine hydroxylase (VTA^TH^) neurons promote wakefulness (Yu et al., 2018), whereas VTA γ-aminobutyric acid (VTA^Vgat^) or VTA glutamic acid decarboxylase 67 (VTA^Gad67^) neurons induce sleep (Eban-Rothschild et al., 2016; Takata et al., 2018). A subset of VTA γ-aminobutyric acid (GABA)–somatostatin (VTA^Vgat-Sst^) cells sense stress and drive non–rapid eye movement sleep (NREMs) and REM sleep (REMs) (Yu et al., 2022). Transient stress enhances the activity of VTA^Vgat-Sst^ cells for several hours, allowing them to persistently exert their sleep effects. Lesioning of VTA^Vgat-Sst^ cells abolished SDS-induced sleep (Yu et al., 2022).

In this regard, it is logical to assume that activation of VTA^Vgat^ neurons after SDS induces sleep and allows animals to recover from stress. However, it is not clear whether the benefits of sleep can be replicated in AD. Is this function impaired in AD and can it exacerbate the anxiety state? Therefore, in this study, we analyzed the activity of VTA^Vgat^ neurons after APP/PS1 mice experienced SDS. We sought to clarify whether activation of VTA^Vgat^ neurons could alleviate anxiety in APP/PS1 mice.

## Materials and methods

### Animals

The experiments were conducted according to the ARRIVE guidelines and approved by the Experimental Animal Ethical Committee of Tongji Hospital affiliated with Huazhong University of Science and Technology. Male APP/PS1 mice aged 6–7 months and retired CD-1 mice aged 6–8 months were obtained from Huafukang Biotechnology Co., Ltd., Beijing, China. All mice were housed in a controlled temperature and humidity facility with access to food and water *ad libitum* and a 12-h light/dark cycle. Prior to the experiments, the mice were allowed to acclimatize to the new environments for at least 1 week. A total of 10 APP/PS1 mice and 5 CD-1 mice were used in the experiments. One APP/PS1 mouse died during the surgery and was therefore excluded from the final analysis.

### Experimental design

All APP/PS1 mice received bilateral adeno-associated virus (AAV) injection and EEG headmount implantation. After 3 weeks, the mice were randomly assigned to four rounds of experiments: non-SDS, SDS, SDS + VTA^Vgat^ vehicle, or SDS + VTA^Vgat^ activation (deschloroclozapine, DCZ). The control round consisted of a 1-h nondefeat procedure in which the experimental mouse was separated from the CD-1 aggressor by a transparent partition, behavioral tests (before sleep), 4 h of EEG recording, and behavioral tests (after sleep). The SDS round consisted of a 1-h SDS procedure, behavioral tests (before sleep), 4 h of EEG recording, and behavioral tests (after sleep). The vehicle round consisted of a 1-h SDS procedure, behavioral tests (before sleep), and administration of the vehicle solution, followed by 4 h of EEG recording and behavioral tests (after sleep). The DCZ round consisted of a 1-h SDS procedure, behavioral tests (before sleep), and administration of DCZ, followed by 4 h of EEG recording and behavioral tests (after sleep). Each mouse had a 9-day interval between each round. After a lapse of 9 days since the last round, 5 out of 9 APP/PS1 mice underwent the SDS procedure while 4 underwent the SDS-control procedure. They were sacrificed 30 min after the procedure for subsequent cFOS immunostaining.

### Stereotactic injection of AAV

Stereotaxic injection of AAV was performed as previously described (Yu et al., 2022). In brief, the mice were anesthetized with inhaled isoflurane (1.5%), and after the loss of the tail-clamp reflex, the head was fixed in a stereotaxic frame. The skull was exposed and cleaned with ethanol. A mixture of 200 nl pAAV-VGAT1-Cre-WPRE (titer: 1.12E+13 v.g./ml) and 200 nl pAAV-EF1a-fDIO-hM3D(Gq)-mCherry (titer: 5.17E+12 v.g./ml) (OBiO Technology, China) was injected bilaterally into the VTA (−3.52 mm AP, ±0.35 mm ML, and − 4.25 mm DV relative to the bregma) through a 10 μL Hamilton syringe at a rate of 0.1 μL/min. Ten minutes after injection, the needle was slowly withdrawn. Subsequently, the EEG electrodes were implanted on the mouse. Experiments started 3 weeks after placement to allow for the expression of the virus and recovery from surgery.

### EEG electrode implantation and signal acquisition

After the AAV injection mentioned above, a headmount (8,201, Pinnacle, USA) was implanted on the skull surface with super glue and dental cement. After recovering from the surgery, the mice were caged singly in a Plexiglas barrel. The mice were allowed to acclimate to the headmount for 3 weeks. The EEG signals were amplified and captured by Sirenia Acquisition software (Pinnacle, USA). The wake, NREMs and REM states were analyzed by a blinded investigator using Sirenia Sleep Pro software (Pinnacle, USA) according to EEG signals and videos taken by a camera (Zhao et al., 2012; Xie et al., 2018).

### Drug administration

According to a previous report (Nagai et al., 2020), 100 mg DCZ (HY-42110, MCE, China) was dissolved in 1 ml DMSO solution and prepared as a 100 mg/ml storage solution in saline. To effectively activate designer receptors exclusively activated by designer drugs (DREADDs) in the mouse brain, DCZ (100 μg per kg) was intraperitoneally injected before the EEG monitor. The vehicle solution was saline containing 2% DMSO.

### Behavioral tests

The open-field test (OFT) was performed to examine anxiety-like behavior as previously described (Cao et al., 2021). In brief, the mice were gently introduced to the open field and allowed to explore the area at liberty for 10 min. Behavioral trajectories were captured and analyzed using the Any-Maze behavioral tracking system (Stoelting, USA).

An elevated plus-maze (EPM) was performed following the OFT to examine anxiety-like behavior as previously described (Komada et al., 2008). In brief, the mouse was introduced into the central area of the maze with its head toward the open arm. The mouse was allowed to explore the maze at liberty for 5 min. The moving trajectories were captured and analyzed using the Any-Maze behavioral tracking system (Stoelting, USA). We have set several anxiety parameters as follows:

Non-middle zone index = non-middle zone time in the After-sleep test/(non-middle zone time in the Before-sleep test + non-middle zone time in the After-sleep test).

Close arm index = duration in the close arm in the After-sleep test/(duration in the close arm in the Before-sleep test + duration in the close arm in the After-sleep test).

### Immunofluorescence staining

The mice were transcardially perfused with normal saline, followed by a precooled 4% paraformaldehyde solution. The brains were postfixed with 4% paraformaldehyde for 12 h and then transferred to a 30% sucrose solution. Coronal sections were cut at a thickness of 14 μm using a cryotome (Leica, CM1950, German). The sections were first washed three times with PBS for 10 min each and then permeabilized with immunostaining permeabilization buffer containing Triton X-100 (P0096, Beyotime, China) for 15 min, followed by 15 min in immunostaining blocking buffer (P0260, Beyotime, China). The sections were then incubated with primary antibody (rabbit anti-cFOS, 1:500, CST, USA) at 4°C for 72 h. After three 10-min washes in PBS, the sections were incubated with the corresponding secondary antibody (FITC-conjugated goat anti-rabbit; 1:400, Jackson Immunoresearch, USA) for 1 h at room temperature, followed by three 10-min washes in PBS before slide mounting. Immunofluorescence images were captured using laser scanning confocal microscopy (Olympus, FV1000, Japan) and then analyzed by a blinded researcher using Fiji software.

### Statistical analysis

The data were analyzed using GraphPad Prism 9.0 (GraphPad Software Inc., La Jolla, CA, USA). A normality test was performed using the *Shapiro–Wilk* test. For analysis of behavioral tests and EEG results, paired *t* tests were used. For analysis of immunostaining results, unpaired *t* tests were used. For correlation analysis, *Pearson* correlation analysis was performed. *p value*s less than 0.05 were considered to be statistically significant.

## Results

### Acute SDS leads to anxiety in APP/PS1 mice

**Figure 1:**
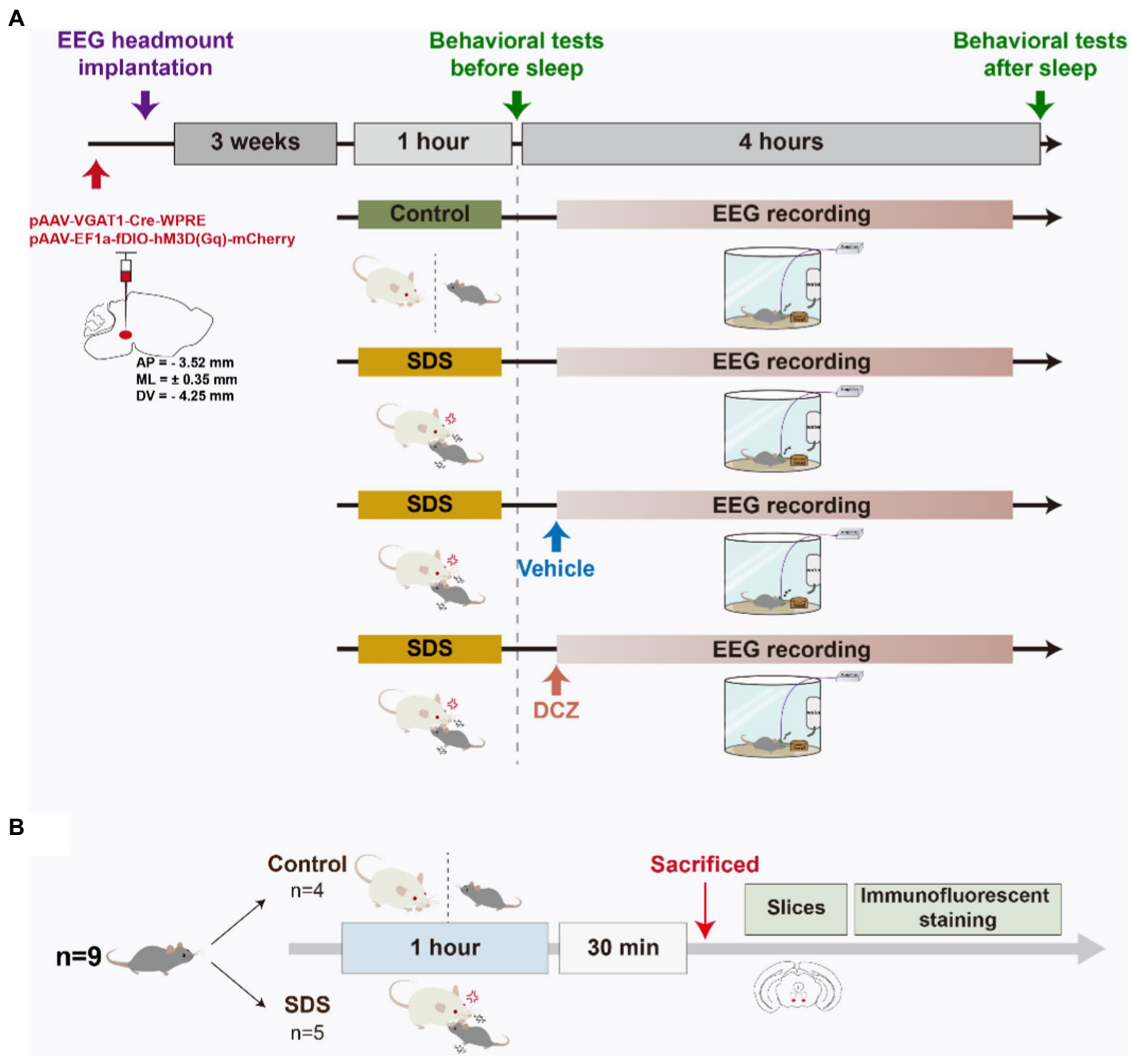
Schematic diagram of the experimental design. **(A)** APP/PS1 mice were stereotaxically injected with pAAV into the VTA, and EEG headmounts were implanted. After 3 weeks of acclimation, the mice randomly underwent four rounds, namely, non-SDS, SDS, SDS + VTA^Vgat^ vehicle, or SDS + VTA^Vgat^ activation. The open field test and elevated plus maze behavioral tests were performed before and after a 4-h EEG recording. Vehicle solution or DCZ was administered before EEG recording in the SDS + VTA^Vgat^ vehicle or SDS + VTA^Vgat^ activation groups, respectively. **(B)** After procedures in A, mice underwent SDS-control and SDS procedure respectively, and were sacrificed 30 min later for tissue immunostaining.

**Figure 2:**
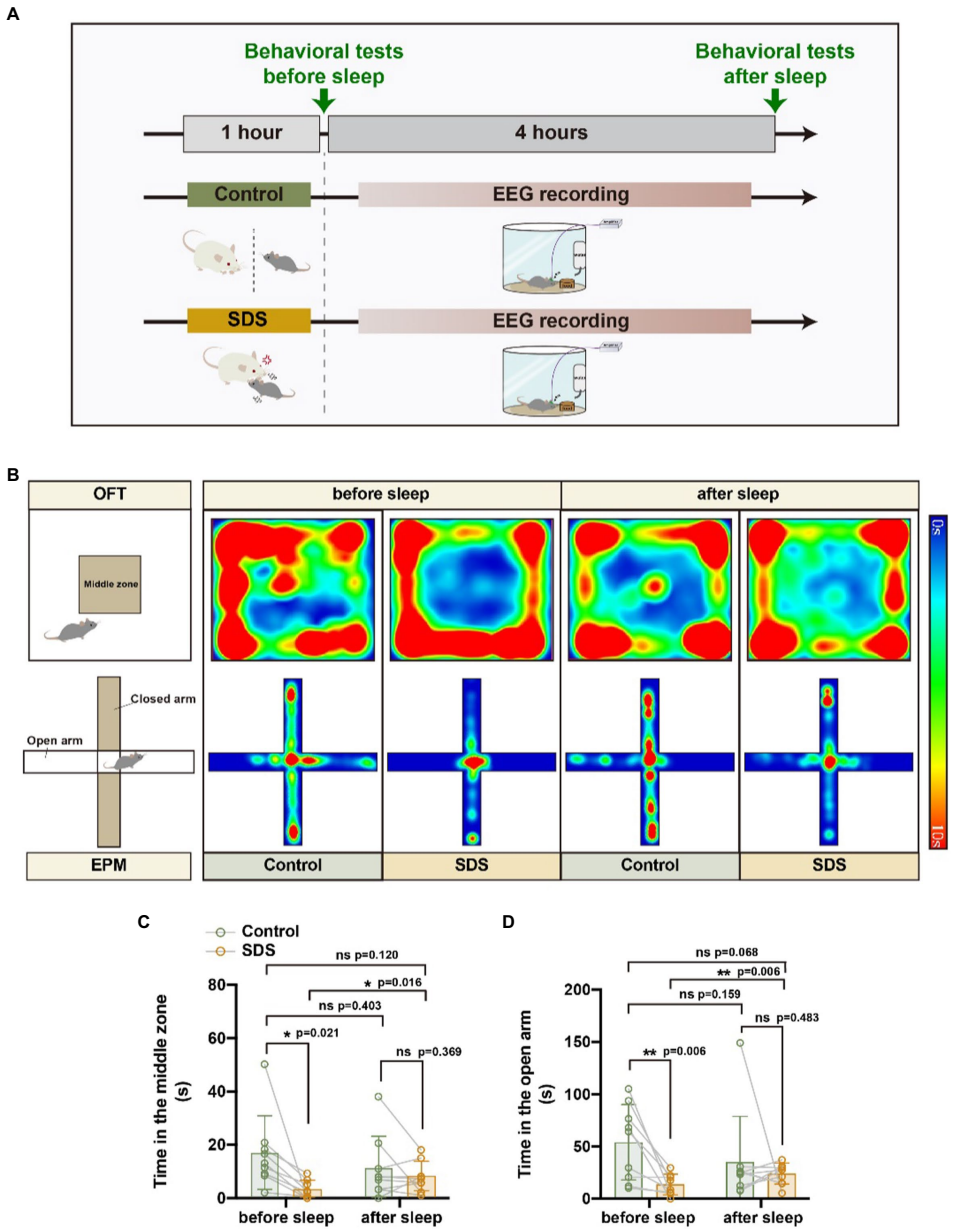
SDS induced anxiety-like behavior, whereas sleep reduced anxiety in APP/PS1 mice. **(A)** Schematic diagram of non-SDS and SDS groups. **(B)** Representative heatmaps of trajectories in OFT and EPM before and after EEG recording in non-SDS and SDS groups. **(C,D)** Quantitative analysis of OFT and EPM in control and SDS groups. *n* = 9, paired *t* test.

We designed an experiment in which the same mice underwent non-SDS, SDS, SDS + VTA^Vgat^ vehicle, or SDS + VTA^Vgat^ activation prior to 4 h of EEG recording. Anxiety-like behavior was assessed by the open field test (OFT) and elevated plus maze (EPM) before and after 4 h of EEG recording. Chemogenetically activated VTA^Vgat^ neurons, pAAV-EF1a-DIO-hM3Dq-mCherry and pAAV-VGAT1-Cre were specifically injected into the VTA of APP/PS1 mice (Figure 1). We first assessed anxiety-like behavior by measuring the time spent in the middle area of the open field and the time spent in the open arms of the elevated plus maze. Mice that experienced SDS for 1 h had rapidly evoked anxiety-like behavior, as seen in the decreased duration spent in the center area or open arms (non-SDS vs. SDS, *p* = 0.021, *t* = 2.881, df = 8 for the middle area; non-SDS vs. SDS, *p* = 0.006, *t* = 3.533, df = 9 for the open arms) (Figures 2A–D).

### SDS promotes sleep and relieves anxiety-like behaviors in APP/PS1 mice

**Figure 3:**
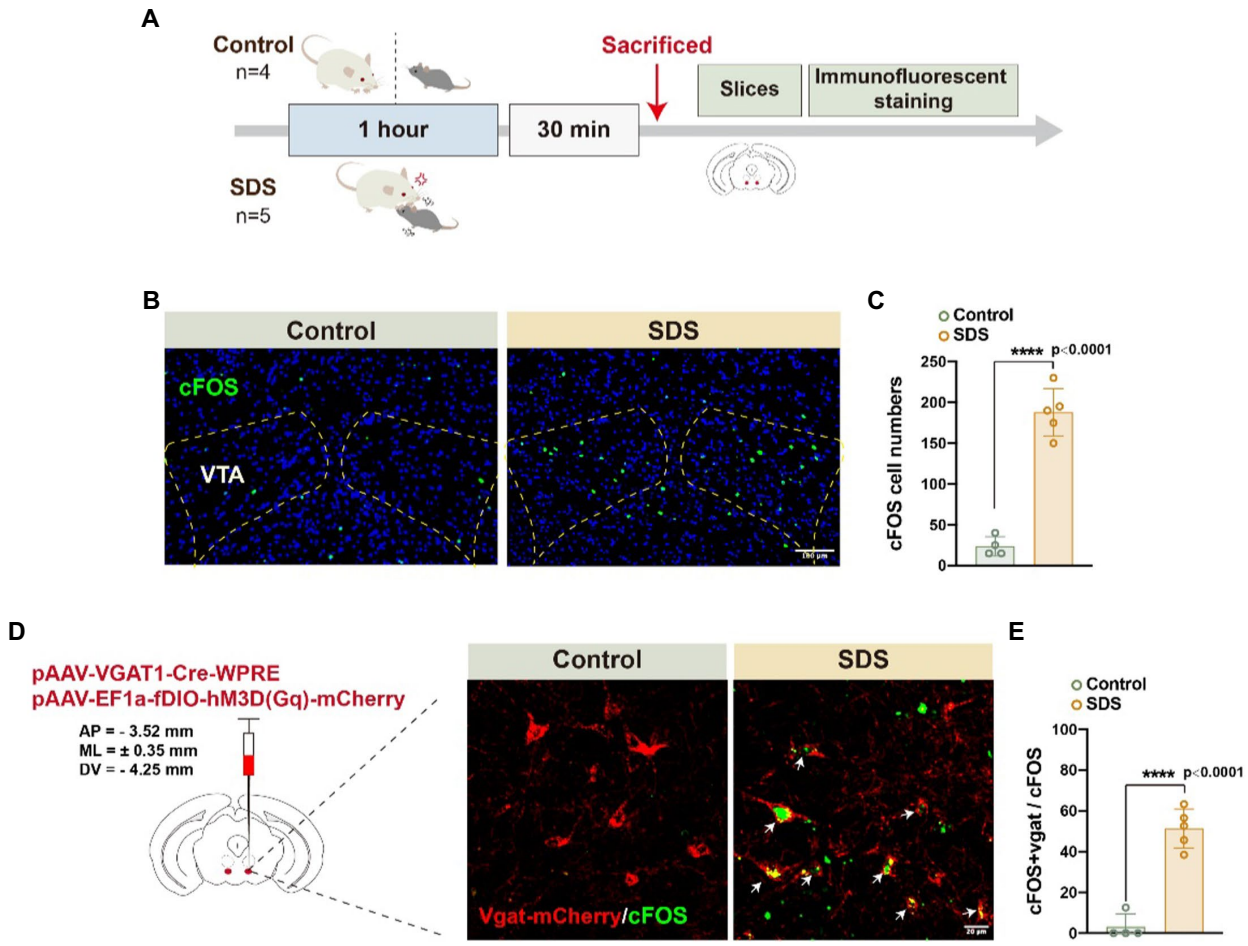
SDS activated VTA^Vgat^ neurons in APP/PS1 mice. **(A)** Schematic diagram of the experimental procedure. The mice were sacrificed 30 min after the 1-h non-SDS (4 mice, control) or SDS (5 mice, SDS). **(B)** Representative images of cFOS expression in the bilateral VTA area in the non-SDS and SDS groups. Scale bar, 100 μm. **(C)** Quantitative analysis of cFOS-positive cell numbers in **(B)**. **(D)** Representative images of Vgat-mCherry and cFOS in VTA area in non-SDS and SDS groups. Arrows point to the colocalization of Vgat and cFOS. Scale bar, 20 μm. **(E)** Quantitative analysis of **(D)** in non-SDS and SDS groups. *n* = 4 for control and *n* = 5 for SDS, unpaired *t* test.

After a 4-h rest, anxiety-like behavior was assessed again by OFT and EPM. We found that the anxiety state of the mice that experienced SDS was significantly reduced, similar to that of the control group, but they still did not appear to have fully recovered to normal (Figures 2C,D). In Figure 2D, it can be observed that the value of p is 0.068 for the comparison of SDS scores after sleep with those of the control group before sleep (*p* = 0.068, *t* = 2.075, df = 9). This indicates a tendency that sleep does not entirely alleviate anxiety. As activation of VTA^Vgat^ neurons induces sleep following SDS stress (Takata et al., 2018; Yu et al., 2022), we further investigated whether VTA^Vgat^ neurons were activated in response to SDS in APP/PS1 mice (Figure 3A). After the SDS protocol, cFOS-positive cells increased prominently in the VTA (*p* < 0.0001, *t* = 10.440, df = 7, *F* = 6.143) (Figures 3B,C). Of these, 51.3% of cFOS-positive cells coexpressed mCherry, indicating that SDS predominantly activates GABAergic neurons in the VTA (*p* < 0.0001, df = 8.615, *f* = 7) (Figures 3D,E).

**Figure 4:**
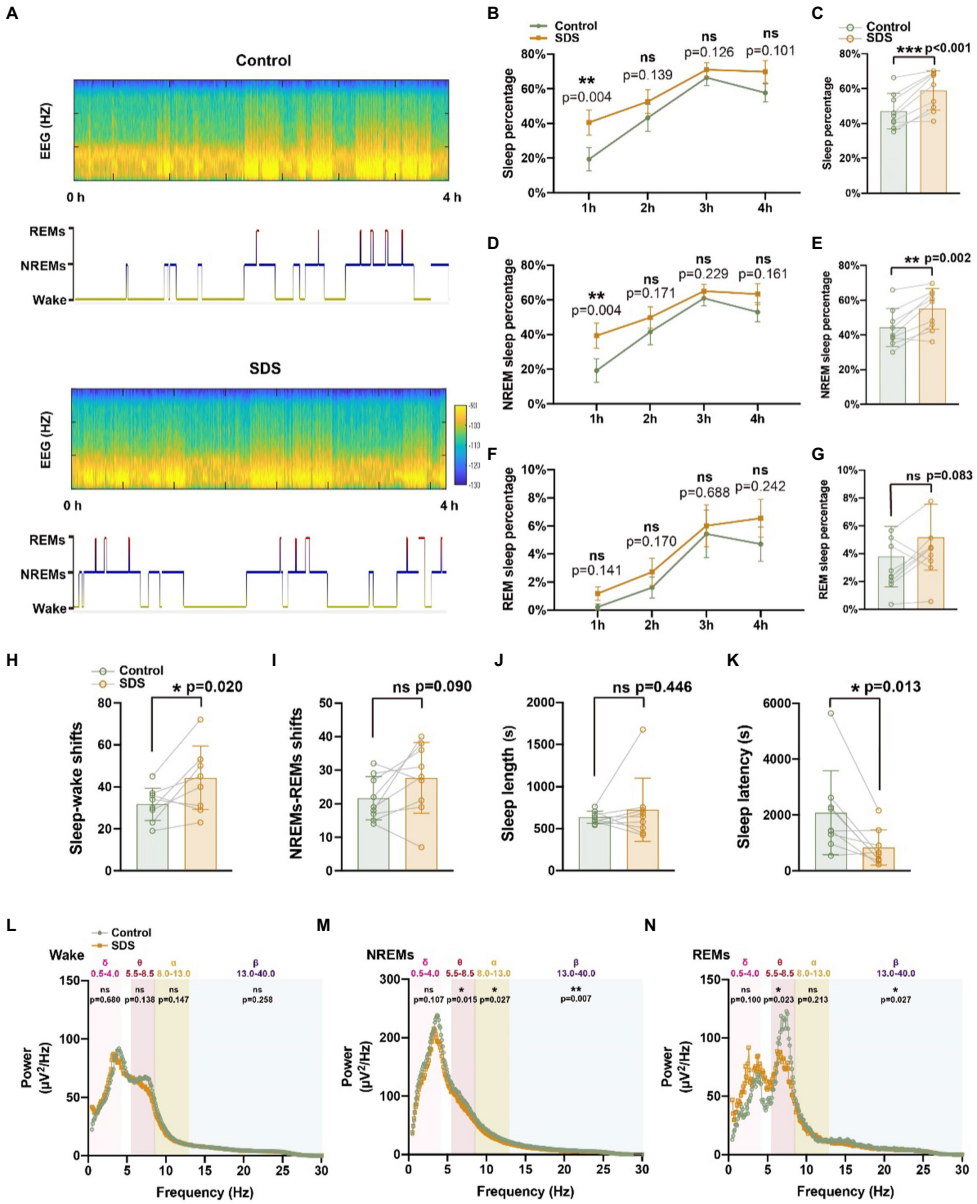
SDS promotes sleep in APP/PS1 mice. **(A)** Representative hypnograms of non-SDS and SDS groups during the 4-h recording. **(B–G)** Quantitative analysis of the percentage of sleep, NREMs, and REMs in each hour **(B,D,F)** and in 4 h **(C,E,G)**. **(H–K)** Quantitative analysis of sleep–wake shifts, NREMs-REMs shifts, sleep length, and sleep latency in non-SDS and SDS groups during the 4-h recording. **(L–N)** Spectral plot in wake, NREMs, and REM phases in non-SDS and SDS groups. *n* = 9, paired *t* test.

Subsequently, we assessed the sleep–wake structure of the mice for 4 h after experiencing SDS or non-SDS (Figure 4A). After SDS, the sleep percentage was increased (*p* < 0.001, *t* = 5.416, df = 8), while only NREMs were enhanced (*p* = 0.002, *t* = 4.454, df = 8) but REMs were not (*p* = 0.083, *t* = 1.980, df = 8) (Figures 4C,E,G). The increase in sleep percentage (*p* = 0.004, *t* = 3.986, df = 8) and NREMs (p = 0.004, *t* = 3.917, df = 8) after SDS was sustained over the 4 h of sleep, especially in the first hour after SDS (Figures 4B,D). Sleep latency was shortened (*p* = 0.013, *t* = 3.198, df = 8), but there was no change in sleep length (*p* = 0.446, *t* = 0.802, df = 8) (Figures 4J,K). Although SDS increased sleep percentage, the sleep and wake shift also increased (*p* = 0.020, *t* = 2.893, df = 8), suggesting that SDS induces poor sleep quality (Figure 4H). EEG spectral analysis revealed that SDS significantly suppressed theta, alpha, and beta power during NREMs, as well as theta and beta power during REMs (Figures 4L–M).

### Activation of VTA^Vgat^ neurons further relieved stress in APP/PS1 mice

Given the restorative function of sleep after SDS, we further explored whether sleep could be promoted by activating VTA^Vgat^ neurons to further relieve stress in APP/PS1 mice. We wondered whether sleep promotion *via* the activation of VTA^Vgat^ neurons could be a strategy to slow anxiety-like symptoms in AD.

**Figure 5:**
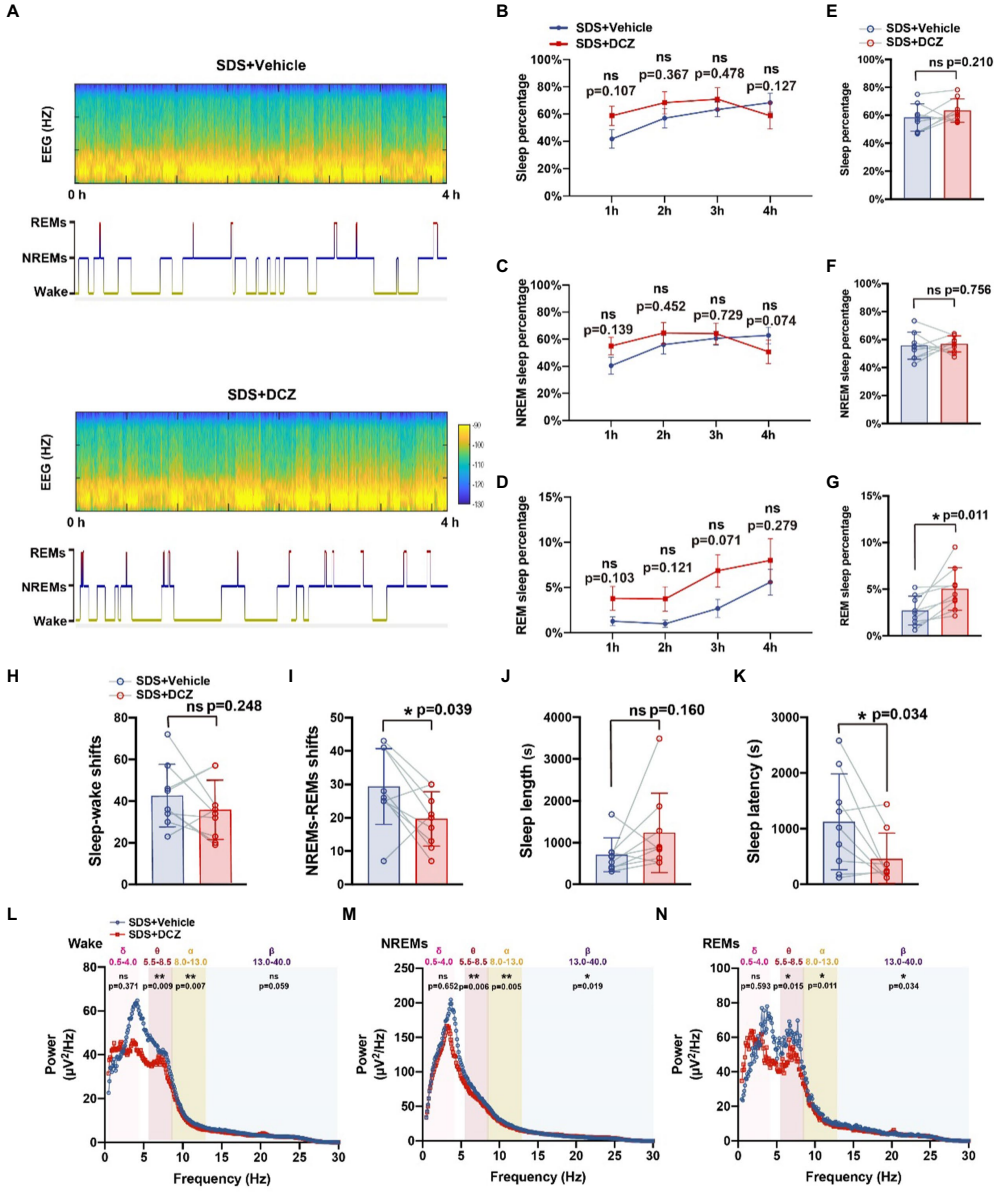
Activation of VTA^vgat^ neurons promotes sleep quality after SDS in APP/PS1 mice. **(A)** Representative hypnograms of SDS + VTA^Vgat^ vehicle or SDS + VTA^Vgat^ activation round during the 4-h recording. **(B–G)** Quantitative analysis of the percentage of sleep, NREMs, and REMs in each hour **(B,D,F)** and in 4 h **(C,E,G)**. **(H-K)** Quantitative analysis of sleep–wake shifts, NREMs-REMs shifts, sleep length, and sleep latency in SDS + VTA^Vgat^ vehicle, or SDS + VTA^Vgat^ activation round during the 4-h recording. **(L–N)** EEG Spectral plot and average wave power in wake, NREMs, and REMs in SDS + VTA^Vgat^ vehicle or SDS + VTA^Vgat^ activation round. *n* = 9, paired *t* test.

We injected AAVs encoding DREADD hM3Dq-mCherry (DIO) and Vgat1-Cre viruses into the VTA to test whether the activation of this VTA subset of neurons could induce sleep (Figures 3D,E). After SDS, VTA^Vgat^ neurons were activated with DCZ. VTA^Vgat^ activation increased the percentage of REMs (*p* = 0.011, *t* = 3.310, df = 8) but not the total sleep percentage (*p* = 0.210, *t* = 1.365, df = 8) and NREM percentage (*p* = 0.756, *t* = 0.322, df = 8). The sleep percentage and NREM percentage in the first 3 h were higher when activated with DCZ but decreased sharply in the fourth hour (*p* > 0.05) (Figures 5B–G). In addition, both the NREMs and REMs shift and the sleep latency (*p* = 0.034, *t* = 2.556, df = 8) were decreased after DCZ activation, suggesting an improvement in sleep quality after the activation of VTA^Vgat^ neurons (Figures 5H–K). EEG spectral analysis revealed that the activation of VTA^Vgat^ neurons after SDS suppressed theta, alpha, and beta power during NREMs and REMs (Figures 5L–N).

**Figure 6:**
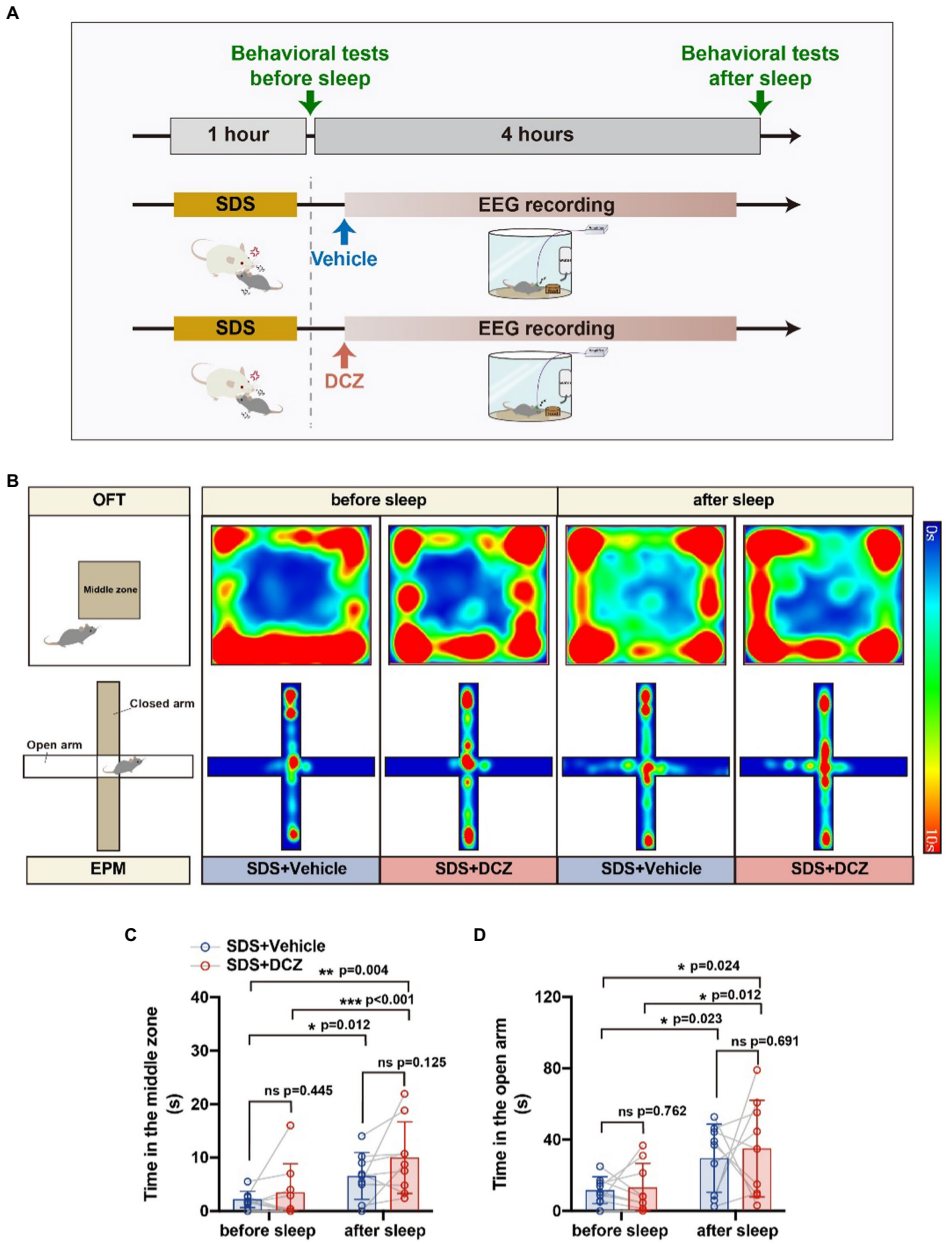
Activation of VTA^Vgat^ neurons further relieved stress in APP/PS1 mice. **(A)** Schematic diagram of SDS + VTA^Vgat^ vehicle or SDS + VTA^Vgat^ activation round. **(B)** Representative heatmaps of trajectories in OFT and EPM before and after EEG recording in SDS + VTA^Vgat^ vehicle or SDS + VTA^Vgat^ activation round. **(C,D)** Quantitative analysis of OFT and EPM in SDS + VTA^Vgat^ vehicle or SDS + VTA^Vgat^ activation round. *n* = 9, paired *t* test.

Behavioral tests showed a significant reduction in anxiety-like behaviors and greater relief of anxiety than without activation of VTA^Vgat^ neurons (in the OFT, vehicle vs. DCZ: *p* = 0.012, *t* = 3.257, df = 8 vs. *p* < 0.001, *t* = 5.178, df = 8; in the EPM, vehicle vs. DCZ: *p* = 0.023, *t* = 2.794, df = 8 vs. p = 0.012, *t* = 3.239, df = 8) (Figure 6). In conclusion, we found that the activation of VTA^Vgat^ neurons after SDS improved sleep quality and further relieved SDS-induced anxiety.

### Sleep structures are correlated with anxiety state

**Figure 7:**
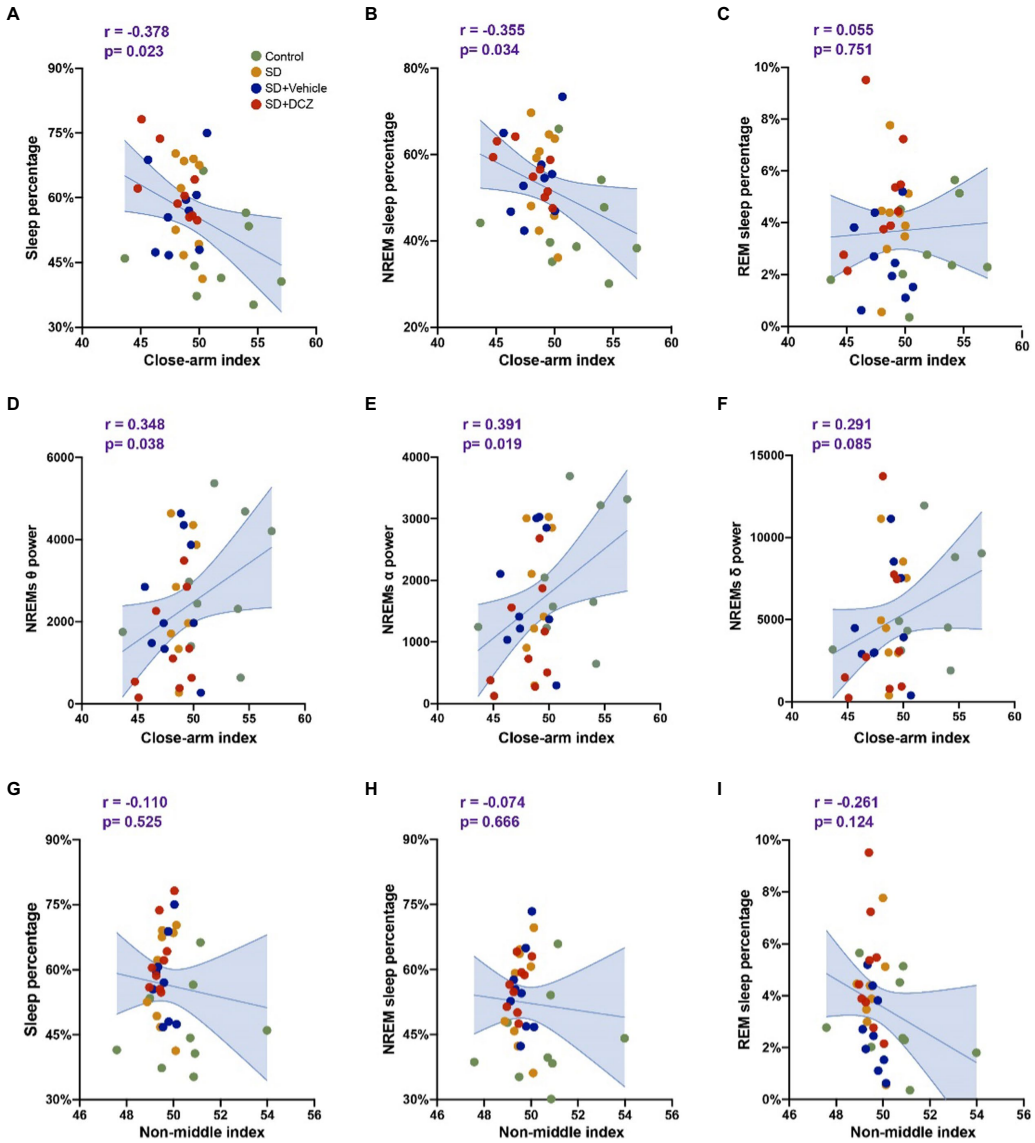
Sleep structures are correlated with anxiety state. **(A–C)** Correlation analysis between close arm index calculated by EPM results and sleep percentage, NREMs percentage, and REMs percentage. **(D–F)** Correlation analysis between close arm index and theta, alpha, and delta powers of NREMs. **(G–I)** Correlation analysis between non-middle index calculated by OFT results and sleep percentage, NREMs percentage, and REMs percentage. Shaded area: 95% confidence interval.

We further analyzed the correlation between sleep structure and anxiety state. We used the proportion of time the mice spent in the closed arm after sleep as the EPM anxiety index and the proportion of time the mice spent in the peripheral area of the open field after sleep as the OFT anxiety index (non-middle index). The percentage of total sleep (p = 0.023) or NREMs (p = 0.034) was found to correlate negatively with the close arm index (Figures 7A,B). Moreover, we found that theta power (*p* = 0.038) and alpha power (*p* = 0.019) during NREMs correlated positively with the close arm index (Figures 7D,F). However, we found no correlation between the percentage of REM sleep or the α power during NREMs and the close arm index (Figures 7C,E). We also did not observe any correlation between sleep structure and non-middle index (*p* > 0.05) (Figures 7G–I, the data of power correlates to non-middle index is not shown).

## Discussion

In the present study, we found that SDS aroused anxiety and activated VTA^Vgat^ neurons in APP/PS1 mice, as previously reported (Lee et al., 2015). SDS prolonged sleep duration but induced poor sleep quality. Nevertheless, sleep after SDS alleviated the anxiety state. Chemogenetical activation of VTA^Vgat^ neurons improved sleep quality and further reduced anxiety in APP/PS1 mice.

Previous studies have shown a complex relationship between Aβ burden and sleep disorders (Sprecher et al., 2015; Winer et al., 2021). Deposition of Aβ is associated with the degree of disruption of NREM slow wave activity (SWA) generation. Furthermore, the reduction in NREM SWA is associated with the impairment of overnight memory consolidation and hippocampal-neocortical memory transformation, which may be an indirect mechanism by which Aβ burden impairs memory in AD. However, sleep clears Aβ from the brain (Xie et al., 2013). Are people with preclinical AD less likely to deal with stress in everyday life and therefore more likely to develop stress-induced cognitive dysfunction? In our study, we tested SDS-induced anxiety using 6- to 7-month-old APP/PS1 mice that typically develop spatial cognitive impairment at 7–8 months of age (Radde et al., 2006; Serneels et al., 2009). We found that, as expected, SDS induced anxiety in APP/PS1 mice. The increase in sleep duration was mainly due to an increase in NREM duration and short sleep latency in APP/PS1 mice. However, SDS induced alterations in sleep architecture, which included increased sleep and wake shift, suggesting that the sleep was fragmented with low quality. Previous studies have shown that changes in sleep architecture are exaggerated in patients with mild cognitive impairment, in whom sleep behavior disorders occur more frequently than normal (Bombois et al., 2010; Musiek et al., 2015). In this regard, although sleep plays a restorative function after SDS, it may not fully relieve anxiety in APP/PS1 mice compared to wild-type mice. However, we did not use wild-type mice to determine the effects of SDS on anxiety, which is a limitation of our study.

NREMs are linked to the brain’s function in clearing Aβ. Conversely, stress causes fragmentation of NREMs, disrupts sleep continuity and can lead to cognitive impairment and anxiety (Benkirane et al., 2022; Bush et al., 2022). In our study, increased NREM duration after SDS may be a key factor in relieving anxiety. Indeed, the proportion of NREMs correlated negatively with anxiety in the results of our correlation analysis. Activation of VTA^Vgat^ neurons further increased REM sleep and shortened sleep latency. Abnormalities in REMs are frequently observed in patients with insomnia, depression, and posttraumatic stress disorder (PTSD) (Maturana et al., 2014; Pace-Schott et al., 2015; Colvonen et al., 2019). In humans, psychosocial stress is one of the main sources of stress. It is uncertain whether activation of VTA^Vgat^ neurons can slow insomnia in APP/PS1 mice. However, it is certain that the activation of VTA^Vgat^ neurons further relieved SDS-induced anxiety. After SDS, theta, alpha, and beta power decreased significantly in NREMs, and these powers were further suppressed after the activation of VTA^Vgat^ neurons. As suggested by numerous published studies, emotional states are associated with oscillatory activity in the brain. Asymmetric theta rhythm is a potential biomarker of depression in humans (Dharmadhikari et al., 2018). Cholinergic theta oscillation appears in human subjects with increased anxiety (Shin et al., 2009). In rodents, the initiation and expression of defensive behavior is characterized by enhanced rhythmicity in the theta range in the medial prefrontal cortex (mPFC), ventral hippocampus (vHPC) and basolateral amygdala (BLA)(Seidenbecher et al., 2003; Tendler and Wagner, 2015; Padilla-Coreano et al., 2016). Theta oscillations reflect synchronized neural firing and are believed to facilitate long-range communication between brain areas involved in the processing and expression of anxiety and fear (Popa et al., 2010; Matulewicz and Ramos-Prats, 2022). Theta rhythms are enhanced during fear response and anxiety states, suggesting that theta rhythm plays a major role in the regulation of these states (Adhikari et al., 2010; Hoeller et al., 2013).

Selective deprivation of REMs, especially for relatively complex tasks, consistently resulted in significant memory impairment (Beaulieu and Godbout, 2000). Prolonged periods of REM deprivation in rodents are associated with a reduction in long-term potentiation (LTP) and an alteration in LTP-related N-methyl-D-aspartic acid receptor (NMDA) receptor subunits in the hippocampus (McDermott et al., 2006; Ravassard et al., 2009), which could explain the memory impairment reported after selective REM deprivation.

VTA GABAergic neurons produce a profound sedative state when artificially activated (Yu et al., 2018), but VTA dopaminergic (VTA*^DA^*) and VTA*^Vglut2^* cells are selectively wake-and REM-active during normal sleep (Eban-Rothschild et al., 2016). Activation of other subtypes of GABA neurons in the VTA, e.g., parvalbumin-and somatostatin-expressing cells, also induced NREM sleep but to a lesser degree when compared to activating the complete set of VTA*^Vgat^* neurons (Yu et al., 2018). VTA^Vgat-Sst^ cells sense stress and drive non–NREMs and REMs through the lateral hypothalamus and inhibit the release of corticotropin-releasing factor in the paraventricular hypothalamus. In our study, we used pAAV-VGAT1-Cre-WPRE and pAAV-EF1a-fDIO-hM3D(Gq)-mCherry to specifically activate VTA^Vgat^ neurons in APP/PS1 mice. Activation of these neurons also has anxiety-reducing effects in APP/PS1 mice.

It is important to note that habituation has an impact on anxiety. In our study, we subjected mice to repeated testing using the same procedure, but we did not observe any anxiety differences in non-SDS mice that were repeatedly tested using OFT and EPM. The role of sleep in memory consolidation or protection against excessive information is not fully understood. In humans, we tend to forget most things and only remember a small fraction of our daily experiences. While sleep may help in eliminating negative stress memories, it is unclear whether fear memories in SDS APP/PS1 mice are consolidated or eliminated. Nevertheless, sleep completely relieves SDS-induced anxiety in APP/PS1 mice, and the activation of VTA^vgat^ neurons alleviates SDS-induced anxiety in APP/PS1 mice.

## Conclusion

In conclusion, we found that acute SDS led to anxiety in APP/PS1 mice, and the activation of VTA^Vgat^ neurons by DCZ alleviated the anxiety. Our results suggest that targeting these neurons could potentially provide a new direction for treating anxiety disorders in AD.

## Data availability statement

The raw data supporting the conclusions of this article will be made available by the authors, without undue reservation.

## Ethics statement

The animal study was reviewed and approved by Experimental Animal Ethical Committee of Tongji Hospital affiliated with Huazhong University of Science and Technology.

## Author contributions

DY, RL, and XL designed the experiment. DY, RL, HH performed the experiment and collected the data. DY and MK were responsible for data analysis. RL prepared the figures. DY, RL, SG, and XL wrote the manuscript. All authors contributed to the article and approved the submitted version.

## Funding

This work was supported by the National Natural Science Foundation of China (81873749 and 81801072).

## Conflict of interest

The authors declare that the research was conducted in the absence of any commercial or financial relationships that could be construed as a potential conflict of interest.

## Publisher’s note

All claims expressed in this article are solely those of the authors and do not necessarily represent those of their affiliated organizations, or those of the publisher, the editors and the reviewers. Any product that may be evaluated in this article, or claim that may be made by its manufacturer, is not guaranteed or endorsed by the publisher.
